# Social and Cultural Contexts of Alcohol Use: Influences in a Social–Ecological Framework

**DOI:** 10.35946/arcr.v38.1.05

**Published:** 2016

**Authors:** May Sudhinaraset, Christina Wigglesworth, David T. Takeuchi

**Affiliations:** May Sudhinaraset, Ph.D., is an assistant professor in the Department of Epidemiology and Biostatics and Global Health Group at the University of California, San Francisco, San Francisco, California. Christina Wigglesworth, M.S.W., L.C.S.W., is a graduate student and David T. Takeuchi, Ph.D., is a professor and associate dean of research, both at the School of Social Work, Boston College, Boston, Massachusetts

**Keywords:** Alcohol consumption, alcohol misuse, morbidity, mortality, risk factors, alcohol-related consequences, social factors, cultural factors, environmental factors

## Abstract

Alcohol use and misuse account for 3.3 million deaths every year, or 6 percent of all deaths worldwide. The harmful effects of alcohol misuse are far reaching and range from individual health risks, morbidity, and mortality to consequences for family, friends, and the larger society. This article reviews a few of the cultural and social influences on alcohol use and places individual alcohol use within the contexts and environments where people live and interact. It includes a discussion of macrolevel factors, such as advertising and marketing, immigration and discrimination factors, and how neighborhoods, families, and peers influence alcohol use. Specifically, the article describes how social and cultural contexts influence alcohol use/misuse and then explores future directions for alcohol research.

The alcohol research literature is overwhelmingly focused on risk factors, from the societal level down to the individual. Worldwide, 3.3 million deaths were attributed to alcohol misuse in 2012 (World Health Organization 2014). Excessive alcohol use is the third leading cause of death in the United States, accounting for 88,000 deaths per year (Centers for Disease Control and Prevention 2014). Globally, alcohol-attributable disease and injury are responsible for an estimated 4 percent of mortality and 4 to 5 percent of disability-adjusted life-years (DALYs) (Rehm et al. 2009). The harmful effects of alcohol misuse are far reaching and range from accidents and injuries to disease and death, as well as consequences for family, friends, and the larger society. Economic costs attributed to excessive alcohol consumption are considerable. In the United States alone, the costs of excessive alcohol use were estimated at $223.5 billion in 2006, or $746 per person (Bouchery et al. 2011). Much of these costs result from a loss in workplace productivity as well as health care expenses, criminal justice involvement, and motor vehicle crashes (Rehm et al. 2009).

This article reviews some of the cultural and social influences on alcohol use and places individual alcohol use within the contexts and environments where people live and interact. This is not an exhaustive review but aims to show the wide range of contexts that may shape alcohol use.

## Disparities in and Influences on Alcohol Use: A Social–Ecological Framework

Alcohol consumption varies across gender and race/ethnicity. Across the world, men consume more alcohol than women, and women in more developed countries drink more than women in developing countries (Rehm et al. 2009). American men are much more likely than women to use alcohol (56.5 percent vs. 47.9 percent, respectively), to binge drink (30.4 percent vs. 16 percent, respectively), and to report heavy drinking (9.9 percent vs. 3.4 percent, respectively) (Substance Abuse and Mental Health Services Administration [SAMSHA] 2013). (Binge drinking is defined here as the number of instances in the past 12 months that women drank 4 or more drinks and men drank 5 or more drinks within a 2-hour period.) Among racial and ethnic groups, Whites report the highest overall alcohol use among persons age 12 and over (57.4 percent). American Indian/Alaska Natives report the highest levels of binge drinking (30.2 percent), followed by Whites (23.9 percent), Hispanic/Latinos (23.2 percent), African Americans (20.6 percent), and Asians (12.7 percent) (SAMHSA 2013). Alarmingly, according to two nationally representative samples, trends in alcohol misuse increased among both men and women and African-American and Hispanic youth over the decade between 1991–1992 and 2001–2002. Rates of dependence also increased among men, young Black women, and Asian men during the same time period (Grant et al. 2004).

Given these trends, it is clear that a better understanding of the underlying social and cultural factors contributing to these disparities is needed. For example, socioeconomic status (SES) indicators (i.e., education, income, and occupation) usually are strong predictors of health behaviors and outcomes and tend to be positively associated with health. People with higher SES tend to drink more frequently than others (Huckle et al. 2010). Among drinkers, low-SES groups tend to drink larger quantities of alcohol (Huckle et al. 2010).

Like other health issues, alcohol use can be linked to a complex array of factors ranging from individual-level (i.e., genetics) to population-level (i.e., cultural and societal factors) characteristics (Berkman et al. 2000; Krieger 2001; Link and Phelan 1995). On a population level, emerging research has documented the relationship between social determinants and health (Berkman and Kawachi 2000; Berkman et al. 2000) and, specifically, the social epidemiology of alcohol use (Bernstein et al. 2007; Galea et al. 2004). Social capital theory suggests that social networks and connections influence health (Berkman et al. 2000). Individuals who have higher levels of social support and community cohesion generally are thought to be healthier because they have better links to basic health information, better access to health services, and greater financial support with medical costs. (Berkman and Kawachi 2000).

This article examines these population-level as well as individual influences through a social–ecological framework, which posits that human health and development occur across a spectrum—from the individual to the macro or societal level (Bronfenbrenner 1994). In the context of alcohol use, individuals are nested within their microsystem (their home, work, and school environments), which is nested itself within the larger community. Macrolevel factors, such as exposure to advertising, may influence family and peer network attitudes and norms, which ultimately affect individual attitudes and behaviors (see figure).

## Societal Influences: Advertising, Marketing, and Social Media

Media exposure helps influence social norms about alcohol through advertising, product placements, and stories in a wide range of sources, including movies, television, social media, and other forms of entertainment. Although alcohol sales and marketing are highly regulated, people are exposed to a wide variety of alcohol and liquor advertisements, especially in the United States. Whether these advertisements directly result in an increase in consumption has been the topic of many public policy debates and much alcohol and consumer research. Recent studies have used robust methodological designs in order to assess the effects of advertisements on alcohol consumption (Grenard et al. 2013; Koordeman et al. 2012). Although longitudinal studies have found that alcohol commercials particularly affected younger adolescents’ propensity to consume alcohol (Grenard et al. 2013), an experimental design randomly assigning college students to alcohol advertisements demonstrated no differences compared with the control group (Koordeman et al. 2012). It is likely that the effects of advertisement differ across age groups and races. The alcohol industry uses complex targeted marketing strategies that focus on African Americans, Latinos, and American Indians, among other demographic groups, such as youth and other ethnic minorities (Alaniz and Wilkes 1998; Moore et al. 2008). Empirical studies show that targeted alcohol marketing results in individuals developing positive beliefs about drinking, and creating and expanding environments where alcohol use is socially acceptable and encouraged (Alaniz and Wilkes 1998; Hastings et al. 2005; McKee et al. 2011). These factors can result in the onset of drinking and binge drinking, and in increased alcohol consumption (Tanski et al. 2015).

Since the introduction of flavored alcoholic beverages in the 1980s, the alcohol industry has engaged in targeted marketing efforts toward youth in general, and especially young women (Mosher and Johnsson 2005). Products with sweet fruity flavors, colorful appearance and packaging, as well as lower alcohol content are designed to appeal to young women. Fruity drinks mask the taste of traditional alcoholic beverages with the sugary flavors of soft drinks (Mosher and Johnsson 2005), making them more palatable for this consumer market. Although the alcohol industry claims that its marketing strategies target adults ages 21–29, products like flavored alcoholic beverages remain attractive to younger drinkers.

Research estimates that 38.5 percent of high school students have used alcohol in the past month, and 20.5 percent of teenagers started drinking before age 13 (Eaton et al. 2012). Approximately 75 percent of high school seniors and 64 percent of high school 10th graders report having experimented with alcohol (Kann et al. 2014). Youth under age 21 see and hear marketing for flavored alcoholic beverages disproportionally on a per capita basis compared with adults (Jernigan et al. 2005), and a disproportionate number of youth consume alcoholic beverages (Mosher and Johnsson 2005). Furthermore, youth exposed to alcohol advertisements tend to drink more on average than their peers who were exposed to less intensive alcohol-related marketing (Snyder et al. 2006). Specifically, the authors found that each additional advertisement viewed by youth increased the reported number of drinks consumed by 1 percent.

Alcohol marketing also can lead to youth and young adults developing alcohol brand preferences (Albers et al. 2014; Ross et al. 2015), which can influence their reports of alcohol consumption (Roberts et al. 2014). For example, youth reported on average 11 more drinks per month when responding to an online survey that used brand-specific measures compared with a survey using more general alcohol measures (Roberts et al. 2014). The relationship between alcohol brand receptivity and alcohol brand consumption also has been linked to whether and when adolescents begin to binge drink (Morgenstern et al. 2014).

Increased use of social media for alcohol marketing has paralleled changes in communication methods among adolescents and college-age youth (Hoffman et al. 2014). Marketing techniques for a wide range of products reflect studies that online platforms are likely to influence adolescent behaviors (Cook et al. 2013). Social media venues are most widely used by youth, with 92 percent of teens reporting being online daily and 24 percent online “almost constantly” (Lenhart 2015). Social-networking sites such as Twitter, Instagram, and Facebook feature alcohol-related marketing. One study found that by 2012, there were more than 1,000 alcohol-related sites on Facebook alone (Nhean et al. 2014). Alcohol use increases with the number of online peer ties and greater peer density, a measure of interconnectedness in the social network (Cook et al. 2013). Despite self-imposed regulations aimed at preventing underage youth from accessing alcohol advertisements on social media, more than two-thirds of advertisements on YouTube are accessible to youth under the legal drinking age (Barry et al. 2015).

Racial and ethnic minorities, especially those living in African-American communities, are likewise exposed to targeted alcohol beverage advertisements (Wilson and Till 2012). African Americans account for 13 percent of the U.S. population, but they purchase 67 percent of all malt liquor sold (Miller Brewing Company 2000). Malt liquor generally has higher alcohol content, is less expensive, and is sold in larger volumes than other beers and ales, and African Americans are exposed to more malt liquor advertisements than other groups. Billboards and other advertisements for malt liquor are disproportionately found in neighborhoods with higher percentages of African Americans, and rap music lyrics frequently mention malt liquor (Herd 2013; McKee et al. 2011). When examining alcohol advertising in newspapers, Cohen and colleagues (2006) found that there were more alcohol-related ads in newspapers targeted to African-American readers compared with newspapers with a more general readership. Kwate and Meyer (2009) found a correlation between problem drinking among African-American women and exposure to alcohol advertisements, suggesting that as ad exposure increased, so did alcohol consumption.

These findings, however, must be interpreted with caution, as it is difficult to determine whether advertisements directly result in increased alcohol consumption. To begin with, a variety of marketing strategies including distribution, product development, pricing, and targeted marketing all may affect links between advertising and consumption (Alaniz and Wilkes 1998; Roberts et al. 2014). For example, Molloy (2015) found that after controlling for targeting, only moderate advertising effects are seen, despite the strong correlations between alcohol advertising and drinking among youth. It also is unclear which aspects of online social media advertisements are related to the observed correlations. Research shows that drinkers like advertising about alcohol more than nondrinkers do, respond neurologically to the advertising more intensively than nondrinkers do, and may recall the advertising more clearly (Snyder et al. 2006), making it harder to distinguish among the specific mechanisms behind the observed relationships. As a result, making causal statements about alcohol use and marketing is problematic because the temporal order between using alcohol and seeing advertisements is not frequently established (Snyder et al. 2006).

Despite these challenges, it is important to develop new strategies to systematically examine the impact of advertising and marketing on alcohol use among different populations. For example, researchers might continue to compare marketing and advertising strategies within specific neighborhoods to more fully understand targeted marketing’s influence on alcohol use. Further research and evaluation studies also are needed that can help establish whether and how advertising and marketing can lead to alcohol use in vulnerable and disadvantaged populations.

## Influences From Discrimination

A number of social and cultural factors predict increased alcohol use, including discrimination and its related stigma. The role of discrimination and stress in health-related risk behaviors, including alcohol use, is well established (Dawson et al. 2005; Hatzenbuehler 2009; Paradies 2006). The stress and coping framework frequently is applied to explain the influence of discrimination and stigma on health (Krieger 1999; Pascoe and Smart Richman 2009; Walters et al. 2002). This long-held theory posits that people consume alcohol to cope with the stress of their daily lives, including work-related stressors and racial and ethnic discrimination (Conger 1956).

Discrimination is seen as a key social stressor that elicits a physiological response, including elevated blood pressure and release of stress hormones (Williams and Mohammed 2009), which may have lifelong deleterious effects, including increased alcohol use (Pascoe and Smart Richman 2009). Self-reported unfair treatment and racial discrimination has been linked to higher alcohol use among Asian Americans (Chae et al. 2008; Gee et al. 2007; Yoo et al. 2010) and Latinos (Mulia et al. 2008).

The picture is less clear among African Americans. Although similar positive associations have been found between level of discrimination and alcohol use in this population (Boynton et al. 2014; Gibbons et al. 2004; Mulia et al. 2008), other recent studies (Kwate and Meyer 2009) among African-American adults have found no relationship between high levels of racial discrimination and heavy and episodic drinking. However, Borrell and colleagues (2007) did report an association between discrimination and past-year alcohol use. The mixed results among African Americans may relate more to SES than to discrimination. Past studies suggest that African Americans with higher levels of education were more likely to report experiencing discrimination, whereas the opposite was true among Whites (Borrell et al. 2007; Krieger et al. 1998). This may be because better educated African Americans find themselves in situations in which they may be exposed to discrimination, or they may be more acutely aware of how subtly it can be expressed. Whites of lower SES may be in the minority and therefore may be more likely to report experiencing discrimination. This may explain the mixed results found in this particular population segment, as socioeconomic position actually may mute the effects of discrimination on alcohol use. Further research is needed to examine these potential mechanisms and other underlying factors that interact with racial discrimination to influence and alcohol use and misuse among minorities.

Another group that may be at particular risk for alcohol problems stemming from their experiences with discrimination are those in the lesbian, gay, bisexual, and transgender (LGBT) community, who experience high levels of discrimination related to sexual orientation and gender identification (Krieger and Sidney 1997). One study found that more than two-thirds of LGBT adults experienced discrimination, and individuals who reported discrimination based on race, gender, and sexual orientation were almost four times more likely to use alcohol and other substances (McCabe et al. 2010). This suggests that future studies and public health interventions should focus not only on racial and gender discrimination, but also sexual orientation and gender identification.

## Immigration-Related Influences

Societal influences can shape drinking behavior among immigrants to the United States. In 2010, nearly 40 million people, or 13 percent of the U.S. population, had been born in another country—the largest absolute number of U.S. immigrants ever and the highest proportion who are foreign born since the 1920s (Grieco et al. 2012). With wide diversity among immigrants in terms of national origin, language, religion, and social class, and with even more reasons for and processes of migration than ever before (Dubowitz et al. 2010), it is no surprise that the evidence on alcohol consumption among immigrants is similarly complex.

Immigration may influence alcohol consumption and its consequences in at least two ways. The first theory suggests that immigrants encounter difficulties and hardships as they transition into a new society and culture (Berry 1997). Hardships include the stress of experiencing new environments and cultures; living in poor neighborhoods; finding good, secure jobs in safe work environments; encountering few opportunities to enhance income or wealth; and engaging with fewer and smaller social networks that may otherwise offer instrumental and emotional support. It also is possible that immigrants may not become fully integrated into American society because of experiences with discrimination and obstacles in social mobility (Unger et al. 2014). Because these factors are associated with alcohol consumption and problems, immigrants may consume more alcohol (Unger et al. 2014). As they become settled in the new society, this consumption pattern decreases (Bui 2012). A second hypothesis posits that alcohol consumption increases the longer immigrants live in a new location (Lee et al. 2013). Over time, immigrants may learn the behaviors and adapt the lifestyles often associated with alcohol consumption in American society (i.e. experience acculturation) (Caetano 1987; Vaeth et al. 2012).

Strong evidence indicates that norms in countries of origin have long-term effects on the drinking patterns of immigrants (Cook et al. 2014). Recent immigrants generally have lower rates of alcohol consumption and excessive drinking than other U.S. residents (Brown et al. 2005; Szaflarski et al. 2011). Available reviews find that acculturation leads to more alcohol consumption among immigrants, including Latinos (Valencia and Johnson 2008; Zemore 2007). Higher acculturation is associated with higher odds of drinking and heavier drinking among Latino women (Zemore 2007). The findings for Latino men appear less clear cut, with high acculturation tied to greater likelihood of drinking but not a definitive pattern for problem drinking.

Studies are beginning to recognize the importance of premigration factors, including levels of alcohol use before migration as well as the cultural influences of countries of origin (Sanchez et al. 2014; Walsh et al. 2014). One study (Sanchez et al. 2014) among Latinos found that Latino men had higher levels of alcohol use before immigration, with steeper declines postmigration compared with Latino women. This finding suggests that future studies may need to focus on trajectories of alcohol use to address alcohol prevention efforts. Moreover, retaining culture of origin also has been shown to have protective influences for alcohol use (Schwartz et al. 2012), including protective family and traditional values.

Timing also may be critical in understanding how immigration is associated with alcohol consumption. Age at immigration can be seen as the developmental context of people’s experiences when they first arrive in the United States. This context helps to shape language use, heterogeneity of social networks, and schooling. The social institutions that affect people’s lives vary by age of immigration (Fuligni 2004; Rumbaut 2004). The number of social groups and institutions, such as schools, clubs, friendship networks, and family ties, geared toward supporting children to integrate into their new society is far greater than those available for adults (Takeuchi et al. 2007). These social groups, in turn, offer children greater access to the opportunity structures in a new culture. Conversely, immigrant children may have a larger set of social groups available to them than older immigrants. As a result, they also could experience a greater amount of negative stressors and influences that could lead to detrimental social and health outcomes as they mature. Immigrants who move to the United States at younger ages may be at risk for behaviors like alcohol use and misuse because they have the potential to be involved in social networks that may offer greater access and opportunity to engage in these behaviors, as well as lower levels of parental attachment (Hahm et al. 2003; Vaeth et al. 2012).

A recent study found that Mexican immigrants who come to the United States before age 14 have higher alcohol consumption rates than those who are older when they immigrate (Reingle et al. 2014). Immigrants who come at a younger age have alcohol consumption patterns similar to their U.S.-born counterparts. The study by Reingle and colleagues also shows that immigrants who arrive when they are younger than 14 and who live beyond the U.S.–Mexico border region have much higher rates of alcohol use than immigrants in the border region. This particular finding suggests that where immigrants live is another social context worth further investigation.

## Community Influences

The literature on community influences on alcohol use focuses primarily on environmental aspects, such as neighborhood characteristics and opportunities for alcohol purchasing and consumption. For example, one study found that individuals who lived in a neighborhood with a poorly built environment, characterized by inferior building conditions, housing, and water and sanitation indicators, were 150 percent more likely to report heavy drinking compared with those living in better built environments (Bernstein et al. 2007). Other studies have examined the spatial epidemiology of neighborhoods regarding alcohol availability, individual consumption, and community disorganization and violence (Cohen et al. 2006; LaVeist and Wallace 2000; Scribner et al. 2000; Shimotsu et al. 2013; Theall et al. 2011). Spatial relations between alcohol outlets and individual consumption also may be a key to explaining differential rates in alcohol use across racial/ethnic groups. A number of studies suggest that minority communities have higher concentrations of liquor stores than White communities (Alaniz and Wilkes 1998; LaVeist and Wallace 2000; Pollack et al. 2005; Romley et al. 2007; Treno et al. 2000), potentially increasing access to alcohol among minority populations (Freisthler et al. 2015; Scribner et al. 2000). Moreover, living in a disadvantaged neighborhood at an early age has long-term effects. Childhood exposure to violence leads to increased exposure to delinquent peers and alcohol use (Trucco et al. 2014). In another study, realizing how easy it is to get alcohol, witnessing neighborhood drug dealing, and seeing peers drink were all associated with increased alcohol use (Chung et al. 2014).

Relating neighborhood characteristics to alcohol use risk is useful for public health program planning because it allows policymakers and programmers to understand how changing structural-level factors of the built environment may affect health risk behaviors, including alcohol use. However, methodological challenges remain when analyzing the impact of complex community factors on individual behaviors. Such factors include social stratification (i.e., the probability of living in certain neighborhoods, which is higher for certain types of persons) and social selection (i.e., the probability that drinkers are more likely to move to certain types of neighborhoods). It remains unclear whether neighborhood disadvantage causes alcohol problems, and whether frequent drinkers are in fact usually more attracted to certain neighborhoods (i.e., self-selection). These challenges limit the interpretation of research on community-level effects. Some studies have attempted to address these issues using propensity matching and time-sensitive indicators (Ahern et al. 2008). Future studies should take these challenges into consideration and address subgroup differences in alcohol use norms across race/ethnicity and gender.

## Cultural Norms

Cultural norms and beliefs are strong predictors of both current drinking and frequent heavy drinking (Brooks-Russell et al. 2013; Caetano and Clark 1999; LaBrie et al. 2012; O’Grady et al. 2011; Paschall et al. 2012). Across race and ethnicity, African Americans and Latinos report more conservative attitudes toward drinking compared with Whites (Caetano and Clark 1999; LaBrie et al. 2012). These more conservative norms may be associated with lower drinking rates among African Americans and Latinos compared with Whites (SAMHSA 2013). Few studies have examined diversity within racial and ethnic groups such as Latinos, Blacks, and Asians, limiting our ability to meet the needs of specific subpopulations. Some studies suggest that alcohol-related problems differ substantially across Latino subgroups, including higher rates of alcohol abuse and dependence among Mexican-American and Puerto Rican men compared with Cuban Americans and Central and South Americans (Caetano et al. 2008). These findings may best be explained by considerable differences in cultural norms, especially the cultural beliefs regarding appropriate alcohol use (Greenfield and Room 1997; LaBrie et al. 2012). For example, some scholars explain heavy-drinking patterns among Latino men through the concept of machismo*,* which has been a significant cultural influence for generations and remains integral to Latino male identity (Dolezal et al. 2000). Machismo suggests that Latino men attempt to appear strong and masculine because of cultural values, and drinking greater amounts of alcohol further exemplifies their masculinity. More recently, scholars have commented that concepts like machismo cannot account for the complexity of Latino drinking behavior (Caetano 1990).

Asians, on the other hand, generally are thought to have higher abstention rates compared with other racial and ethnic groups, especially when they are integrated within their ethnic cultures (Cook et al. 2012). One measure of the retention of ethnic values and cultural norms is generation status. That is, the longer immigrants have lived in the United States, the more likely they are to acculturate to the cultural norms of their destination community (Berry et al. 2006). Lower levels of ethnic identity may be one explanation for these differences across Asian subgroups. Japanese Americans, Filipino Americans, and Korean Americans often have been in the United States longer than other Asian subgroups, such as Cambodians, Thais, and Vietnamese, and also report higher levels of alcohol use compared with other Asian Americans and Asian immigrants (Iwamoto et al. 2012). Ethnic identity may promote stronger family values and traditional ties, leading to lower levels of alcohol use. Moreover, Asian-American adolescents who have a high attachment to family or who share their family’s negative attitudes toward drinking are less likely to consume alcohol (Hahm et al. 2003).

Cultural norms also vary by context and place. Some alcohol researchers have used multilevel approaches to distinguish among the causal effects of individual and neighborhood-level norms. For example, Ahern and colleagues (2008) found that neighborhood norms against drunkenness were a more robust and stronger predictor of binge drinking than permissive beliefs about it held either by the individual or family and friends. If an individual lived in a neighborhood that frowns on binge drinking, that individual was less likely to drink, even if he or she believed it acceptable to do so. This was particularly true for women, suggesting gender norms around alcohol use may be a factor.

Specifically, past studies found that gender differences in alcohol use may reflect the greater social stigma directed at women who drink. This seems to be more pronounced in certain cultures. Caetano and Clark (1999), for example, found stronger gender norms related to alcohol use in Latino cultures compared with the United States (Kulis et al. 2012). This results in greater gender differences in alcohol use among Latinos compared with other U.S. populations, with recent trends suggesting similar levels of binge drinking between men and women in Western cultures (Iwamoto et al. 2012). This may reflect changing beliefs about gender and social status. Although traditionally perceived as a “masculine” behavior, binge drinking is now more acceptable among women in certain cultures that foster more balanced gender roles (Lyons and Willott 2008).

## Family and Peer Influences on Adolescent and Young Adult Drinking

Some of the strongest influences on adolescent drinking behavior come from the people that youth spend the most time with: family and friends. Studies have found that higher levels of alcohol use among parents and peers is associated with increased alcohol use among adolescents and young adults (Cruz et al. 2012; Dawson 2000; Mares et al. 2011; Osgood et al. 2013; Trucco et al. 2014; Varvil-Weld et al. 2014; Wallace et al. 1999; Walsh et al. 2014; Williams and Smith 1993). Developmentally, people’s social contexts shift from the family unit during childhood to focus more on their peers and their schools during adolescence. Reflecting this, parental alcohol use seems to exert a greater influence before age 15 and diminishes over time (Dawson 2000).

Conversely, family support, bonding, and parental monitoring is associated with lower alcohol use (Bahr et al. 1995; White et al. 2006) and social networks and social support also have protective effects (Ramirez et al. 2012). For example, one study that assessed the effects of leaving home and attending college found that although the transition overall was associated with higher levels of alcohol use, young people with fewer friends who use alcohol reported higher levels of religiosity. Higher parental monitoring also protected against alcohol and marijuana use (White et al. 2006). Moreover, higher levels of familism (values that place family needs over individual needs) and being in a nuclear family served as protective factors among adolescents (Ewing et al. 2015).

Peer norms play an important role at this life stage (Jackson et al. 2014). By the late adolescent period, parental influences related to alcohol use are small compared with peer influences (Schwinn and Schinke 2014; Zehe and Colder 2014). Much of the focus on peer influences has highlighted the risk networks associated with alcohol use. Peer pressure (Studer et al. 2014), peer alcohol norms (Varvil-Weld et al. 2014), and socializing with substance-using peers (Patrick et al. 2013) were associated with alcohol misuse and binge drinking. Studies note that leaving the home environment, entering college, and joining Greek organizations increased alcohol use as a result of more socially permissive norms around drinking (Scott-Sheldon et al. 2008; White et al. 2006).

More recent studies have attempted to assess the synergistic influence of peers and families. Whereas the majority of studies on peers have focused on the negative consequences of social networks, research shows that greater parental support and monitoring can lead to prosocial peer affiliations (Williams et al. 2015). One study found that protective influences in parental domains can moderate the negative effects of negative peer influences among Latino college students (Varvil-Weld et al. 2014). In particular, maternal communication resulted in less alcohol use; conversely, maternal permissive norms and peer norms were associated with more alcohol use. Greater parental disapproval toward alcohol use is associated with lower involvement in peer networks that use alcohol, less peer influence to use, and greater self-efficacy and stronger negotiation skills to avoid alcohol (Nash et al. 2005). Interventions aimed at establishing and fostering conservative peer norms were found to be more effective than individual resistance training (Hansen and Graham 1991), whereas multilevel interventions incorporating peers, families, and communities are known to be effective among adolescents (Chapman et al. 2013; Perry et al. 2002; Toumbourou et al. 2013).

Existing successful interventions to reduce alcohol use include incorporating culturally sensitive delivery models, such as employing community health workers among Latino populations (Ornelas et al. 2014) and using Web-based interventions to change norms (Patrick et al. 2014). In a recent review, *Familias: Preparando la Nueva Generación,* a culturally grounded intervention for parents to support Mexican-heritage youth, showed reductions in parental drinking (Williams et al. 2015). Because past studies show that parents may potentially moderate negative peer influence, fostering synergistic solutions between multiple contexts should be a priority (Ewing et al. 2015).

## Directions for Future Research

This article highlights examples of how societal factors, cultural norms, neighborhoods, and social contexts may be associated with alcohol misuse. Certain gaps in the literature clearly remain. Methodologically, these findings should be interpreted with caution, because it is difficult to distinguish between and among societal and community-level influences. Future studies should use advanced statistical methods such as multilevel modeling techniques, based on theoretical and conceptual approaches in population health. In addition, longitudinal data will help support causal hypotheses and relationships.

Risk and protective factors, prosocial peer affiliations, and synergistic relationships between social contexts are worth further research. Among immigrants, retaining the cultural values of the country of origin has shown to have protective influences on alcohol use, and this finding should be incorporated into future interventions for immigrant populations. Focusing on risk and protective factors will help inform future programs addressing alcohol initiation, specifically helping parents and communities understand how they may influence alcohol use among adolescents and young adults.

Alcohol research should also more actively acknowledge new social contexts among youth culture. A better understanding of the influence online social networking sites and new media have on alcohol use is particularly important among adolescent populations, and this should be explored more fully in future studies.

Developmentally appropriate strategies are needed to delay initiation of alcohol use, because the family environment may be less influential compared with the influence of peers, social norms, and media among older adolescents and young adults. Future interventions should focus on multiple levels of societal environments, from the community to the individual level.

Finally, given the changing demographic landscape of the United States, including a larger and more diverse immigrant population, interventions and treatment options should also reflect the growing needs of certain groups. However, studies have found that focusing only on changing social norms is insufficient, and that broader interventions that influence multiple levels of an individual’s environment, such as family and schools, may have greater impact. Alcohol education programs need to also address individual intent and motivations while offering personalized feedback and protective behavioral strategies (Patrick et al. 2014). Public health and treatment programs need to be culturally sensitive, paying particular attention to cultural factors such as ethnic identification and orientation.

## Figures and Tables

**Figure:**
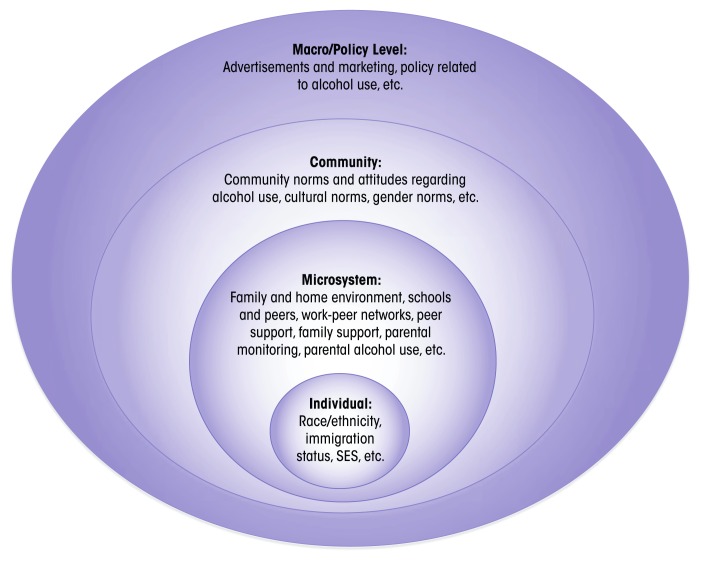
A social–ecological framework for explaining influences on alcohol use. Individual-level factors that influence alcohol use are nested within home, work, and school environments, which are nested within the larger community. Macro-level factors, such as exposure to advertising, may influence family and peer network attitudes and norms, which ultimately affect individual attitudes and behaviors.
